# Recent Advances in Soil Health: Influences of Organic Carbon and Microbiota

**DOI:** 10.3390/biology14050500

**Published:** 2025-05-04

**Authors:** Audrius Kačergius, Audrius Gegeckas, Renata Gudiukaitė

**Affiliations:** 1 Lithuanian Research Centre for Agriculture and Forestry, Kedainiai Distr., LT-58344 Akademija, Lithuania; 2 Institute of Biosciences, Life Sciences Center, Vilnius University, Sauletekis av. 7, LT-10257 Vilnius, Lithuania; audrius.gegeckas@gf.vu.lt (A.G.); renata.gudiukaite@gf.vu.lt (R.G.)

As the deterioration of the overall condition of soils is a growing concern, more and more attention is being paid in the scientific literature to the search for and analysis of measures that can improve the ability of the soil to accumulate organic carbon and increase the availability of nutrients to plants. When assessing the overall condition and health of the soil, chemical indicators usually dominate, although signals indicating the importance of soil biodiversity are already sufficient. In this case, the quantitative parameters of soil microbial communities are very important, also in addition to the taxonomic composition and the functional capabilities that often depend on it, which determine the condition and sustainability of the soil. In this Special Issue, we invited scientists to publish the latest research results in the field of soil health, including studies on soil microbial communities and linking them to the overall analysis of soil condition. Soil organic carbon is very important because it is the main component of soil organic matter, improving soil structure, nutrient availability, and water retention, which are essential for plant growth and productivity. Reduced organic carbon levels result in less nutrient availability for plants, which in turn reduces productivity. Without the sequestering effect of organic carbon, soils become susceptible to erosion and desiccation [1,2]. In general, maintaining sufficient soil organic carbon is essential for sustainable agriculture and the preservation of the surrounding environment.

Another major problem is the processes in the soil that lead to changes in vegetation cover, and in some cases the loss of vegetation cover leading to desertification. In this respect, comprehensive knowledge of soil microorganisms is essential in the fight against vegetation loss and desertification. Soil microorganisms, by breaking down organic matter, improve fertility and thus help to re-establish vegetation in impoverished areas. In addition, microorganisms release substances that can bind soil particles together, thereby strengthening soil structure and reducing erosion. Microbial activity increases organic carbon, and some microbes establish symbiotic relationships with plants, thus helping to absorb nutrients and increase plant resistance to adverse conditions [3]. Changes in vegetation cover have a significant impact on soil microorganism communities, their diversity, and their abundance. Different plants contribute/excrete different amounts and types of organic matter to the soil, which affects the nutrient supply of microorganisms. For example, vegetation diversity can increase the amount of organic carbon and nitrogen in the soil, supporting a more complex microbial network [4]. Plants with extensive and strong root systems improve soil structure by creating channels for air and water movement, which in turn supports microbial activity and improves soil health [5]. Vegetation also influences soil temperature and moisture levels, which are important factors for microbial growth and activity. Seasonal changes in vegetation also lead to changes in microbial community composition [6].

Soil microbial carbon use efficiency (CUE) is an important enough parameter to determine soil status. A higher CUE means that more carbon is converted into microbial biomass rather than being lost as CO_2_ through respiration. This helps to store more C_org_ in the soil, which contributes to the formation of stable soil organic matter, improving soil structure, water retention and overall soil health. Soils with more organic carbon have better moisture and nutrient retention, making them more resilient to extreme weather conditions such as drought [7,8]. Understanding and developing soil CUE can contribute to more sustainable agricultural practices and better ecosystem management.

This Special Issue of Biology “Recent Advances in Soil Health: Influences of Organic Carbon and Microbiota” contains three original research papers and two review articles focusing on the issues described above. Of particular interest is a review article on intracellular enzymes secreted by soil microorganisms, which play a key role in organic matter decomposition and carbon (C), phosphorus (P) and nitrogen (N) cycles and serve as an indicator of soil health and fertility [9]. This article has countless hits and an incredibly high citation count for the short time that has passed since publication. Another extremely important indicator is soil microbial carbon use efficiency (CUE), which is the ratio of the carbon available for microbial growth to the carbon consumed by microorganisms, which is reviewed in a second paper [10]. The research papers address current issues related to soil health: vegetation cover degradation, the impact of organic fertilizers and bioinputs on soil quality agrochemical and microbiological indicators, and changes in microorganism communities linked to rhizosphere components [11,12,13].

Finally, we would like to thank all the authors who submitted manuscripts, as well as the reviewers for the revisions they carried out to improve the quality of this Special Issue and the staff of the *Biology* Editorial Office for their wide-ranging support.

## References

[1] Haddaway N.R., Hedlund K., Jackson L.E., Kätterer T., Lugato E., Thomsen I.K., Jørgensen H.B., Söderström B. (2015). What are the effects of agricultural management on soil organic carbon in boreo-temperate systems?. Environ. Évid..

[2] Keller A.B., Handler S. (2024). Soil Organic Carbon in Temperate Managed Ecosystems: A Primer. Technology Transfer.

[3] Pan C., Yuan F., Liu Y., Yu X., Liu J. (2025). Soil bacterial and fungal diversity and composition respond differently to desertified system restoration. PLoS ONE.

[4] Iqbal S., Begum F., Nguchu B.A., Claver U.P., Shaw P. (2025). The invisible architects: Microbial communities and their transformative role in soil health and global climate changes. Environ. Microbiome.

[5] Huang P., Shi H., Jiang L., Zhu D., Zhou Z., Hou Z., Ma X. (2025). Soil microbial community and influencing factors of different vegetation restoration types in a typical agricultural pastoral ecotone. Front. Microbiol..

[6] Wu H., Xiong D.-H., Xiao L., Zhang S., Yuan Y., Su Z.-A., Zhang B.-J., Yang D. (2018). Effects of vegetation coverage and seasonal change on soil microbial biomass and community structure in the dry-hot valley region. J. Mt. Sci..

[7] Dang R., Liu J., Lichtfouse E., Zhou L., Zhou M., Xiao L. (2024). Soil microbial carbon use efficiency and the constraints. Ann. Microbiol..

[8] Xiao K.-Q., Liang C., Wang Z., Peng J., Zhao Y., Zhang M., Zhao M., Chen S., Zhu Y.-G., Peacock C.L. (2024). Beyond microbial carbon use efficiency. Natl. Sci. Rev..

[9] Daunoras J., Kačergius A., Gudiukaitė R. (2024). Role of Soil Microbiota Enzymes in Soil Health and Activity Changes Depending on Climate Change and the Type of Soil Ecosystem. Biology.

[10] Yu W., Sheng L., Wang X., Tang X., Yuan J., Luo W. (2025). Soil Microbial Carbon Use Efficiency in Natural Terrestrial Ecosystems. Biology.

[11] Du S., Xie H., Zhang G., Qiao F., Geng G. (2025). Improvement Effects of Different Afforestation Measures on the Surface Soil of Alpine Sandy Land. Biology.

[12] Sivojienė D., Masevičienė A., Žičkienė L., Ražukas A., Kačergius A. (2024). Soil Microbial Community Structure and Carbon Stocks Following Fertilization with Organic Fertilizers and Biological Inputs. Biology.

[13] Kuźniar A., Włodarczyk K., Jurczyk S., Maciejewski R., Wolińska A. (2023). Ecological Diversity of Bacterial Rhizomicrobiome Core during the Growth of Selected Wheat Cultivars. Biology.

